# The diagnostic reliability and validity of noninvasive imaging modalities to assess leptomeningeal collateral flow for ischemic stroke patients: A systematic review and meta-analysis

**DOI:** 10.1097/MD.0000000000025543

**Published:** 2021-05-07

**Authors:** Chaohua Cui, Ye Hong, Jiajia Bao, Li He

**Affiliations:** Department of Neurology, West China Hospital, Sichuan University, Chengdu, China.

**Keywords:** image, leptomeningeal collateral flow, reliability, stroke, validity

## Abstract

Leptomeningeal collateral flow (LMF) is associated with infarct area and clinical outcome for ischemic stroke patients. Although LMF can be detected by multiple imaging methods, but their diagnostic performance is uncertain.

The aim of this study was to evaluate the diagnostic validity or reliability of noninvasive image methods in assessing LMF.

Databases included PubMed, Web of Science, Embase, and Cochrane Library.

Original observational cohort studies.

Ischemic stroke patients.

Different noninvasive image methods to assess LMF.

Newcastle–Ottawa Scale to evaluate the quality of the studies; forest plot to show pooled results; *I*^2^ and Egger test to evaluate the heterogeneity and publication bias.

Thirty of the 126 selected studies were eligible. For CT angiography, the interobserver agreement ranged from 0.494 to 0.93 and weighted kappa was 0.888; for patients receiving thrombolysis or endovascular treatment, 0.68 to 0.91; 0.494 to 0.89 for the 2-point system, 0.60 to 0.93 for the 3-point system, 0.68 to 0.87 for the system of >4 points; area under the curve (AUC) was 0.78. For perfusion computed tomography (CTP), the interobserver agreement ranged from 0.724 to 0.872; for patients receiving thrombolysis or endovascular treatment, 0.74 to 0.872; 0.724 for the 2-point system, 0.783 to 0.953 for the 3-point system; the intraobserver agreement was 0.884; AUC was 0.826. For MRI-fluid attenuated inversion recovery (FLAIR), the interobserver agreement ranged from 0.58 to 0.86; for patients receiving thrombolysis or endovascular treatment, 0.75 to 0.86; 0.86 for the two-point system, 0.77 to 0.87 for the system of more than 5 points; AUC was 0.82.

No pooled data of CTP and FLAIR. The difference cohort study had difference bias. The unpublished data were not included.

CT angiography is a good tool for assessing LMF. CTP shows a good validity and reliability, but its diagnostic value needs more evidence. FLAIR is a good modality to assess LMF. These image methods had better validity and reliability to evaluate LMF of patients receiving thrombolysis or endovascular treatment than all ischemic stroke patients.

## 1 Introduction

For ischemic stroke patients, efficient leptomeningeal collateral flow (LMF) can improve the clinical outcome by reducing the volume of penumbra.^[1–3]^ The LMF condition can be used to evaluate the ischemic stroke patient's outcome and prognosis.^[3]^

Presently, multiple imaging techniques are available to examine LMF. The application of digital subtraction angiography (DSA), the golden standard to assess LMF, is limited by its invasiveness and complexity, as well as the patient's or the hospital's condition.^[4]^ In clinical practice, noninvasive methods with simple operation and wide application, such as CT angiography (CTA) and MRI-fluid attenuated inversion recovery (FLAIR), have also been introduced to assess LMF.^[3,5–9]^ Normally the effectiveness of an imaging method should be evaluated by its validity and reliability. The validity indicates the veracity of a method. The reliability, which refers to the agreement reached between different observers or at difference times by the same observers, indicates the stability and credibility of a method. However, the effectiveness of these noninvasive methods in assessing LMF was uncertain,^[5–9]^ and the different grading systems of these methods further intensify the uncertainty.^[5–9]^

A review published in 2012 showed the difference in reliability among the different imaging methods.^[10]^ But the study had not evaluated the validity of different image methods.

To find out a better tool to assess LMF, We conducted a systematic review and meta-analysis using the recently published studies evaluating the diagnostic validity and reliability of multiple imaging methods.^[3,5–9]^

## 2 Methods

The study design, data collection and analysis, and result report all followed the MOOSE Guidelines.

### 2.1 Search strategy

We searched the databases (PubMed, Web of Science, Embase, and Cochrane Library) for studies published before October 1, 2019. The key words of search were ([leptomeningeal collateral] AND stroke). Then we obtained articles evaluating the diagnostic validity, reliability, and consistency of LMF-assessing methods.

### 2.2 Study selection

**Figure 1:**
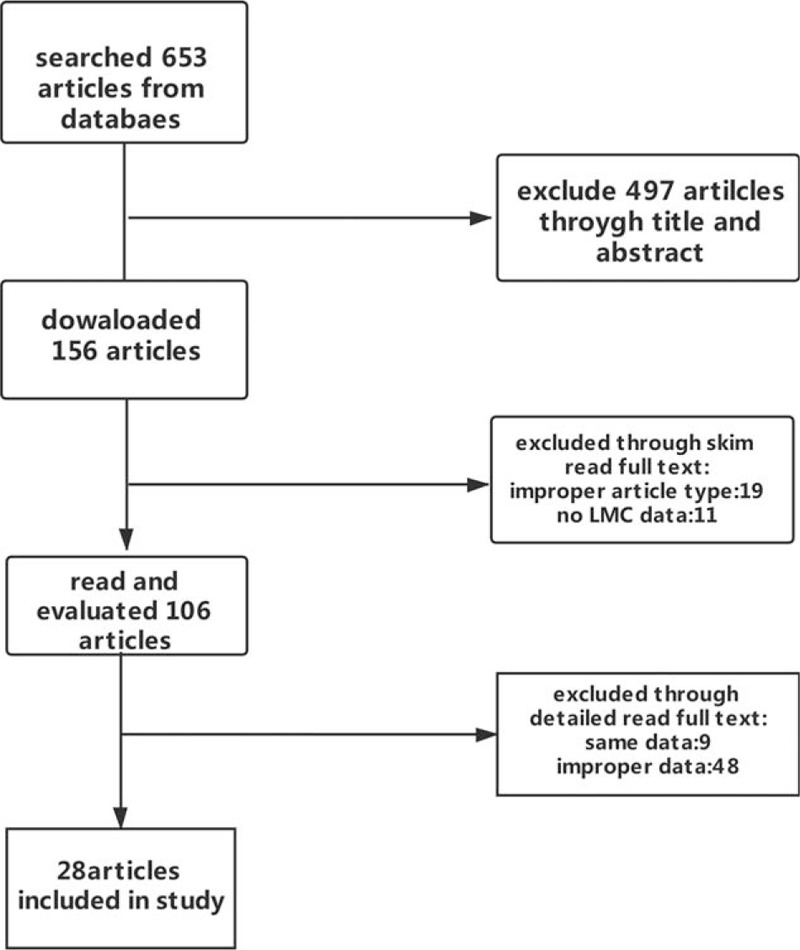
Flowchart of included studies.

We (CC and YH) independently screened the articles. We downloaded the articles after reading the titles and abstracts. After reading full text, we further excluded the articles of the unwanted type or with unrelated outcomes. From the references of the articles, we supplemented the qualitative articles. The search process was shown in Figure 1. We did not exclude the studies for language and geographic reasons.

### 2.3 Inclusion criteria

Inclusion criteria were: original observational cohort studies; studies on ischemic stroke patients; studies on diagnostic validity or reliability of different imaging methods to assess LMF; studies demonstrated validity by area under curve (AUC) and reliability by kappa coefficient.

### 2.4 Exclusion criteria

Exclusion criteria were: studies with the number of patients <20; animal studies; letters, meeting reports, unpublished studies.

### 2.5 Data collection and quality evaluation

We (CC and JB) independently extracted the data, including name of the author, date of publication, region, number, age and type of subjects, study design, imaging techniques, grading system, value of AUC, and value of kappa. We independently evaluated the quality of the studies by Newcastle–Ottawa Scale and gave a score ranging from 1 to 5 points. A higher score was given to the study with a scientific design, appropriate inclusion and exclusion criteria, well-handled confounding factors and bias, rationally assessed risk factors and outcome. The disagreement on the same study was solved through discussion.

The study was performed in accordance with the Declaration of Helsinki and the ethical standards of the institutional and/or national research committee. The study was approved by the Ethics Committee of West China Hospital, Sichuan University.

### 2.6 Statistical analysis

The diagnostic validity was evaluated using the extracted values of AUC and 95% confidence interval (CI) (available data). The reliability was evaluated with the extracted kappa and 95% CI (available data). The reliability was explained by the interobserver agreement (the consistency of observation results of different observers for the same group of objects in the same situation) and the intraobserver agreement (the consistency of observation results of the same observer for the same group of objects in the same situation). When assessing intraobserver agreement, the value of intraclass correlation coefficient was equal to the value of kappa.^[11]^ So we extracted value of intraclass correlation coefficient as value of kappa when a study showed intraobserver agreement.

We pooled a weighted kappa value and 95% CI using Fleiss et al's methods.^[11]^ We demonstrated the kappa value of each eligible study and pooled kappa value in a forest plot. We evaluated the reliability by Landis and Koch’ criteria^[12]^ (value of kappa: <0, poor; 0.1–0.2, slight; 0.21–0.40, fair; 41–0.60, moderate; 0.61–0.8, substantial; 0.81–1, almost perfect). The heterogeneity of the studies was evaluated by *I*^2^. When *I*^2^ value was not >0.5, these studies were considered to have no significant heterogeneity. When available, the different grading system and subgroup of modalities, data of stroke patients receiving thrombolysis or endovascular treatment were further analyzed. All the data were processed by StataMP 14 software (Stata Corp, College Station, TX).

## 3 Results

### 3.1 Study identification and description

Among the 653 searched articles from the databases, we downloaded 156 articles. Finally, 28 eligible articles were selected for this meta-analysis. The search process was showed in Figure 1. Among these articles, 18 evaluated the reliability or consistency of CTA in assessing LMF; 4 articles evaluated CTP in assessing LMF (Table 1); 5 articles evaluated FLAIR in assessing LMF; 2 articles evaluated other magnetic resonance imaging (MRI) modalities in assessing LMF (Table 2).

**Table 1:** Based on CT assess leptomeningeal collateral flow.

Author	Year	Country	Age	No.	Modality	Design	Type of data	Content of value	Grade	Quality
Miteff et al^[3]^	2009	Australia	74	92	CTA	Prospective	Kappa	Interobserver	2	4
Tan et al^[5]^	2007	USA	68	113	CTA	Retrospective	Kappa	Interobserver	2	4
Liu et al^[7]^	2016	China	59.4	52	CTA	Retrospective	Kappa	Interobserver	2	2
Saarinen et al^[13]^	2014	Finland	68.8	105	CTA	Retrospective	Kappa	Interobserver	5	3
Sundaram et al^[14]^	2017	India	57	65	CTA	Retrospective	Kappa	Interobserver	2	3
Tan et al^[15]^	2009	Canada	70	85	CTA	Retrospective	ICC	Interobserver	4	3
Menon et al^[16]^	2011	Canada	NO	138	CTA	Retrospective	ICC	Interobserver	3	3
Frölich et al^[17]^	2014	Germany	73	82	CTA	Retrospective	ICC	Interobserver	3	3
Zhang et al^[18]^	2018	China	70	158	CTA	Retrospective	ICC	Interobserver	2	4
Zhang et al^[19]^	2016	China	69	80	CTA	Retrospective	ICC	Interobserver	20	2
Agarwal et al^[20]^	2012	UK	71.2	39	CTA	Prospective	Kappa	Interobserver	5	3
Menon et al^[21]^	2013	Australia	57–89	41	CTA	Cohort	Kappa	Interobserver	3	4
Gerber et al^[22]^	2016	Germany	69	93	CTA	Retrospective	Kappa	Interobserver	2	3
Nannoni et al^[23]^	2019	Switzerla	72.3	857	CTA	Prospective	Kappa	Interobserver	4	3
Knauth et al^[24]^	1997	Germany	59.8	21	CTA	Prospective	Kappa	Interobserver	3	3
Kim et al^[25]^	2017	korea	68	104	CTA	Retrospective	Kappa	Intraobserver	5	3
Rusanen et al^[26]^	2015	Finland	68.7	104	CTA	Retrospective	ICC + ka	Intraobserver	5	3
van Seeters et al^[27]^	2015	Netherland	67.2	1374	CTA + CTP	Prospective	AUC	Validity	3	4
Calleja et al^[28]^	2013	Spain	73	54	CTP	Prospective	Kappa	Interobserver	2	4
Kim et al^[29]^	2012	South Korea	63.7	54	CTP	Prospective	Kappa	Interobserver, intraobserver	4	2
Jiang et al^[30]^	2017	Australia	73	270	CTP	Retrospective	Kappa	Interobserver	no	4

AUC = area under curve, CTA = CT angiography, CTP = perfusion computed tomography, ICC = intraclass correlation coefficient.

**Table 2:** Based on MRI assess leptomeningeal collateral flow.

Study	Year	Country	Age	No.	Modality	Design	Type of data	Content of value	Grade	Quality
Haussen et al^[8]^	2013	USA	67.7	49	FLAIR	Retrospectively cohort	Kappa	Interobserver	No	3
Pop et al^[9]^	2016	France	63	89	FLAIR	Retrospectively cohort	Kappa + AUC	Interobserver	6	3
Ahn et al^[31]^	2015	Korea	65.1	35	FLAIR	Retrospectively cohort	Kappa + *r*	Interobserver	8	3
Ichijo et al^[32]^	2015	Japan	79	48	FLAIR	Retrospectively cohort	Kappa	Interobserver	11	3
Kufner et al^[33]^	2015	Germany	71.4	62	FLAIR	Retrospectively cohort	Kappa	Interobserver	2	3
Hernandez-Perez et al^[34]^	2016	Spain	65	25	MRA	Retrospectively cohort	Kappa	Interobserver	2	4
Lou et al^[35]^	2017	China	73.9	55	ASL	Prospective cohort	Kappa	Intraobserver	3	2

ASL = arterial spin-labeling, AUC = area under the curve, FLAIR = MRI-fluid attenuated inversion recovery, MRA = magnetic resonance angiography.

### 3.2 CT

**Figure 2:**
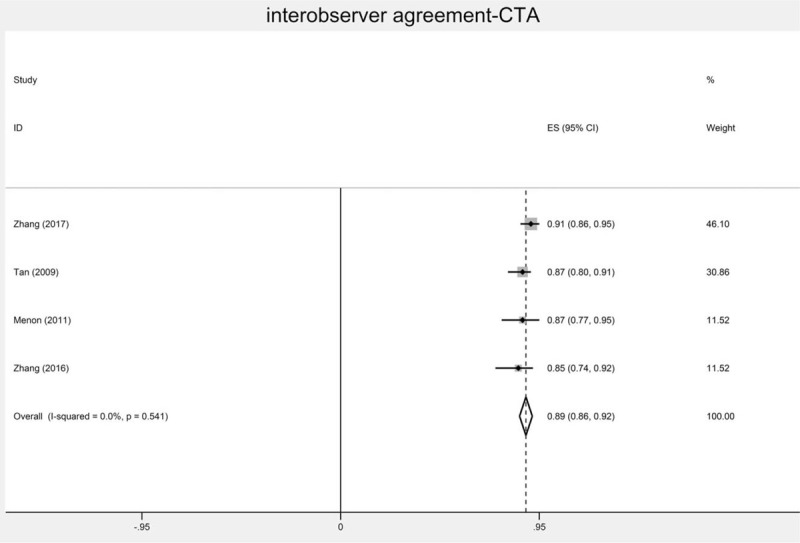
The interobserver agreement of CT angiography methods.

The interobserver agreement of CTA ranged from 0.494 to 0.93.^[3,5,7,13–23]^ The weighted kappa of interobserver agreement in 4^[15,16,18,19]^ eligible studies was 0.886 (Fig. 2). The *I*^2^ was 0, indicating no significant heterogeneity among these four studies. For patients receiving thrombolysis or endovascular treatment,^[13,17,18,22]^ the interobserver agreement of CTA ranged from 0.68 to 0.91. For systems of different grades, the interobserver agreement of CTA ranged from 0.494 to 0.89 for the systems of 2 points^[5,14,22,23]^; the interobserver agreement of CTA ranged from 0.60 to 0.93 for the systems of 3 points^[3,7,16–18,21,24]^; the interobserver agreement of CTA ranged from 0.68 to 0.87 for systems of >4 points.^[13,15,19,20]^ For difference modalities, the interobserver agreement of CTA source image was 0.494; the interobserver agreement of CTA tMIP ranged from 0.669 to 0.87; the interobserver agreement of CTA peak phase was 0.85.^[5,19]^ The intraobserver agreement for CTA ranged from 0.78 to 0.971.^[24–26]^ For patients receiving thrombolysis or endovascular treatment,^[25,26]^ the intraobserver agreement for CTA ranged from 0.87 to 0.91. CTA had an AUC of 0.78, indicating that CTA had better diagnostic performance.^[27]^

The interobserver agreement of CTP ranged from 0.724 to 0.953.^[28–30]^ For patients receiving thrombolysis or endovascular treatment,^[29,30]^ the interobserver agreement of CTP ranged from 0.74 to 0.872. For systems of different grades, the interobserver agreement of CTP was 0.724 for the 2-point system^[28]^; the interobserver agreement of CTP ranged from 0.783 to 0.953 for the 3-point system^[7]^; the interobserver agreement of CTP was 0.884 for the 4-point system.^[29]^ For difference modalities, the interobserver agreement of CTP CBF was 0.828; the interobserver agreement of CTP CBV was 0.783; the interobserver agreement of CTP TTP was 0.942; the interobserver agreement of CTP MTT was 0.886.^[7]^ The intraobserver agreement of CTP was 0.872.^[29]^ The AUC of CTP was 0.826, suggesting that a good diagnostic efficiency.^[7]^

Multi-CT (combined CTA and CTP) had an AUC of 0.85, suggesting its good diagnostic efficiency.^[27]^

### 3.3 MRI

The interobserver agreement of FLAIR ranged from 0.58 to 0.86.^[8,9,31–33]^ For patients receiving thrombolysis or endovascular treatment,^[9,32,33]^ the interobserver agreement of Flair ranged from 0.75 to 0.86. For systems of different grades, the interobserver agreement of FLAIR was 0.86 for the 2-point system^[33]^; the interobserver agreement of FLAIR ranged from 0.77 to 0.87 for the system of >5 points.^[9,31]^ FLAIR showed an AUC of 0.82, indicating a good diagnostic performance of this modality.^[9]^

The interobserver agreement of magnetic resonance angiography (MRA) was 0.93.^[34]^ The intraobserver agreement of arterial spin-labeling (ASL) (multi-delay arterial spin-labeling perfusion imaging) ranged from 0.83 to 0.92.^[35]^

## 4 Discussion

This meta-analysis reviewed the diagnostic performance of different image modalities in assessing LMF. For CT methods, CTA showed an unstable interobserver agreement, a substantial to perfect intraobserver agreement, and a better validity. For systems of different grades, the systems of 3 points or >4 points had a better interobserver agreement than the system of 2 points. For different modalities, tMIP or peak phase modalities had a better interobserver agreement than the source image modalities. CTP had a substantial to perfect interobserver agreement, a perfect intraobserver agreement, and good validity. The systems of 3 points or >4 points had a better interobserver agreement than the 2-point system. For difference modalities, TTP modalities showed a better interobserver agreement than other modalities. Multi-CT had a better diagnostic performance. FLAIR had a substantial to perfect interobserver agreement and good validity. Difference grade system had No significantly different interobserver agreement for FLAIR. MRA had a perfect interobserver agreement and ASL had a perfect intraobserver agreement. When used in patients receiving thrombolysis or endovascular treatment, CTA, CTP, and FLAIR all had a better reliability than used in commonly ischemic stroke patients.

DSA used to be a major method to evaluate LMF, but its clinical use has shrunk due to its invasiveness and complexity. Jansen et al^[6]^ showed that DSA results were not associated with the patients’ clinical outcome, indicating its limitation in clinical use. CT is more widely used for acute ischemic stroke patients because of its high efficiency and universality. Several studies showed that CTA results were positively correlated with ischemic stroke patients’ outcome and prognosis.^[6,23]^ Therefore, CTA may be a better tool for LMF assessment. CTP is another commonly used CT modality to assess LMF. One study concludes that LMF condition of CTP assessment had no significant relationship with ischemic stroke patient's outcome and prognosis.^[28]^ Therefore, the diagnostic value of CTP in LMF assessment needs more evidence. Combined use of CTA and CTP may work. Liu et al and van Seeters T showed the high diagnostic value of multi-CT.^[7,27]^ But more clinical studies are needed to verify the advantages of multi-CT.

Although MRI is often used to evaluate the lesion in acute ischemic stroke patients, it is more frequently applied in ischemic stroke patients of stable stage because it is more time-consuming. FLAIR is a common MRI modality to evaluate LMF. Haussen et al and Karadeli et al showed that FLAIR-detected vascular hyperintensity was correlated with the ischemic stroke patient's LMF status and infarct core.^[8,36]^ Therefore, FLAIR may be a preferable tool for LMF assessment. Although FLAIR and CTA are both preferable tools for LMF assessment, the 2 modalities only have a weak positive correlation.^[36]^ These 2 modalities are based on different imaging mechanisms and applied in different stages, which may partly explain their weak association.^[3,8]^ MRA and ASL are also MRI modalities to assess LMF, and both have a good reliability. But there lacked evidence proving their effect in the stroke patient's prognosis. More studies are needed to verify the advantage of the 2 modalities.

We found that different grading systems had different effects on the modality's reliability. For CTA, the 2-point grading system had a poorer reliability than the other grading system, suggesting that grading system of higher scores could improve the agreement of CTA. Meanwhile, we found the different grading systems did not exert significant effects on the reliability of CTP and FLAIR. The different grading system had difference effect above modalities partly because these modalities had each distinctive imaging mechanism.^[3,7,8]^

When used to evaluate the LMF of patients receiving thrombolysis or endovascular treatment, CTA, CTP and FLAIR all showed a better reliability. The thrombolysis or endovascular treatment could partly recover the blood supply in patients. Hence, compared with other patients, these patients had a more stable blood supply for the lesion,^[37]^ which increased the stability of LMF assessment. In addition, patients receiving thrombolysis or endovascular treatment often had more severe lesions,^[37]^ so these patients’ outcome depend more on larger brain collateral circulation, such as LMF. The above-mentioned 2 reasons indicate that CTA, CTP, and FLAIR are more suitable for patients receiving thrombolysis or endovascular treatment.

Our meta-analysis has several limitations. First, we could not pool data from studies on CTP and FLAIR, which might have affected the statistical accuracy of the results. Second, it is unavoidable that different bias could be found in different cohort studies. But the cohort studies reflecting a real-world condition had a better practical meaning for clinical. In addition, unpublished data and meeting abstracts were not included in this study, which could increase the bias of results.

In summary, CTA is a good tool for LMF assessment, and a grading system of >3 points could have a better reliability. CTP shows a good validity and reliability in evaluating LMF, but the diagnostic value needs more evidence. FLAIR is a good modality to assess LMF for ischemic stroke patients. The above 3 image methods had better validity and reliability to evaluate LMF of patients receiving thrombolysis or endovascular treatment than all ischemic stroke patients. Multi-CT, MRA, and ASL may serve as an optimum choice, but their advantages need more evidence.

## Acknowledgments

The authors thank Professor Guanjian Liu who offered some guide for the methods of pooled data; Dr Yanbo Li who offered some suggestion and guide for the study's design; and MD Shuju Dong who assessed and judged data for the study.

## Author contributions

Professor He and Dr Cui conceived and designed the meta-analysis. Dr Cui, MD Hong and MD Bao screened and collected data for the article. Dr Cui conducted statistical analyses and written the manuscript. All authors reviewed and revised the paper.

**Conceptualization:** Chaohua Cui, Ye Hong, Jiajia Bao, Li He.

**Data curation:** Chaohua Cui, Ye Hong, Jiajia Bao.

**Formal analysis:** Chaohua Cui, Ye Hong, Jiajia Bao.

**Investigation:** Chaohua Cui, Ye Hong.

**Methodology:** Chaohua Cui, Jiajia Bao.

**Writing – original draft:** Chaohua Cui.

**Writing – review & editing:** Chaohua Cui, Ye Hong, Jiajia Bao, Li He.
